# New Biochemical Insights into the Mechanisms of Pulmonary Arterial Hypertension in Humans

**DOI:** 10.1371/journal.pone.0160505

**Published:** 2016-08-03

**Authors:** Renata Bujak, Jesús Mateo, Isabel Blanco, José Luis Izquierdo-García, Danuta Dudzik, Michał J. Markuszewski, Victor Ivo Peinado, Martín Laclaustra, Joan Albert Barberá, Coral Barbas, Jesús Ruiz-Cabello

**Affiliations:** 1 Centre of Metabolomics and Bioanalysis (CEMBIO), Facultad de Farmacia, Universidad CEU San Pablo, Campus Monteprincipe, Boadilla del Monte, Madrid, Spain; 2 Department of Biopharmaceutics and Pharmacodynamics, Medical University of Gdańsk, Al. Gen. J. Hallera 107, 80–416, Gdańsk, Poland; 3 Centro Nacional de Investigaciones Cardiovasculares Carlos III (CNIC), Madrid, Spain; 4 Hospital Clinic Barcelona-IDIBAPS, Barcelona, Spain; 5 CIBERESP, Preventive Medicine and Public Health Department, Facultad de Medicina, Universidad Autónoma de Madrid, Madrid, Spain; 6 Ciber de Enfermedades Respiratorias (CIBERES), Madrid, Spain; 7 Universidad Complutense de Madrid, Madrid, Spain; Instituto de Investigación Sanitaria INCLIVA, SPAIN

## Abstract

Diagnosis of pulmonary arterial hypertension (PAH) is difficult due to the lack of specific clinical symptoms and biomarkers, especially at early stages. We compared plasma metabolic fingerprints of PAH patients (n = 20) with matched healthy volunteers (n = 20) using, for the first time, untargeted multiplatform metabolomics approach consisting of high-performance liquid and gas chromatography coupled with mass spectrometry. Multivariate statistical analyses were performed to select metabolites that contribute most to groups’ classification (21 from liquid in both ionization modes and 9 from gas chromatography-mass spectrometry). We found metabolites related to energy imbalance, such as glycolysis-derived metabolites, as well as metabolites involved in fatty acid, lipid and amino acid metabolism. We observed statistically significant changes in threitol and aminomalonic acid in PAH patients, which could provide new biochemical insights into the pathogenesis of the disease. The results were externally validated on independent case and control cohorts, confirming up to 16 metabolites as statistically significant in the validation study. Multiplatform metabolomics, followed by multivariate chemometric data analysis has a huge potential for explaining pathogenesis of PAH and for searching potential and new more specific and less invasive markers of the disease.

## Introduction

Pulmonary arterial hypertension (PAH) is a heterogeneous disease with multifactorial pathophysiology. PAH is currently classified into various clinical phenotypes, but they all share their severity and progressiveness as common features. The lack of specific clinical symptoms, especially at early stages hinders the diagnosis [1]. PAH, when not diagnosed, can lead to right ventricle failure and consequently to premature death. Additionally, pathomechanisms of PAH remain still not fully understood. Knowledge regarding pathological hallmarks of PAH, such as cell proliferation, apoptosis resistance, vascular remodelling, vasoconstriction and increased angiogenesis, derives mainly from experimental animal models [2,3]. For this reason, new, sensitive and specific markers of PAH in humans are needed to better understand the pathological processes of the disease and consequently improve current diagnosis and treatment. Metabolomics focuses on qualitative and quantitative analysis of low-molecular-weight compounds (metabolites) in various biological samples (plasma/serum, urine, saliva, tissue, exhaled breath) to understand the complex and dynamic responses of living systems to diverse stimuli, such as pathological processes, drug treatments, genetic variability or environmental factors [4,5]. The metabolome, as a final consequence of derangements in genome and proteome is considered to be a link in the genotype-to-phenotype gap. In the area of PAH research, we consider that the application of metabolomics can be a potential tool for understanding its pathogenesis and to find new diagnostic markers. Only a few reports can be found in the literature suggesting the role of metabolic alterations, such as: excessive cellular glucose uptake, glycolytic metabolism, high-density lipoprotein cholesterol and insulin resistance in PAH pathogenesis [6]. One of the most recent studies employed a metabolomics approach to determine metabolic profiles of lung tissue derived from patients with severe PAH [7]. Obviously, analysis of tissue samples provides detection of site-specific metabolite alterations that might be characteristic for disease stage. However, its application in diagnosis and clinical practice is limited due to its invasiveness, while plasma analysis could provide diagnostic markers more readily available to clinicians.

In order to search for potential markers of pathological conditions occurring in PAH, untargeted multiplatform metabolomics, using high-performance liquid and gas chromatography coupled with mass spectrometry (LC-MS and GC-MS), was applied to plasma samples of PAH patients and healthy controls, providing data on the plasma metabolic fingerprint of PAH.

## Materials and Methods

### Study design and samples

This case-control study included 20 patients with confirmed PAH derived from Hospital Clinic in Barcelona and 20 healthy controls. Plasma samples were collected at fasting condition at the same time of the day into EDTA tubes and frozen at -80°C for aproximately 6 months, until metabolomic analysis. The studied groups were matched according to age (*p* = 0.96), body mass index (*p* = 0.87) and sex (*p* = 0.62). Independent recruitment of other additional 20 patients and 12 controls processed in a separated batch and not used in the main analyses allowed external validation. The investigation was carried out in accordance with approval of The ethical committee of clinical investigations in Barcelona (CEIC, the approval number CIF-G-08431173) and the informed consent was signed by each participant of the study. The detailed characteristics of study and validation cohorts are described in S1 and S2 Tables. Plasma was separated from fasting blood samples for metabolic fingerprinting. Metabolomics included liquid chromatography-mass spectrometry (LC-MS) in positive and negative modes and gas chromatography-mass spectrometry (GC-MS).

### Plasma metabolic fingerprinting with HPLC-ESI-QTOF-MS and GC-EI-Q-MS

Sample preparation for LC-MS included deproteinization, centrifugation and filtration. Quality control samples (QCs) were prepared as aliquots of a pool of equal volumes from all samples included in the study, using the same preparation procedures. Samples were analysed by an HPLC system connected to a Q-TOF LC-MS detector. Randomized samples were analysed in two separate runs (for positive and negative modes). Sample preparation for GC-MS involved deproteinization, centrifugation, supernatant’s evaporation and two-step derivatization employing methoxymation and silylation. GC−MS analysis was performed with a gas chromatograph interfaced to an inert quadrupole analyser.

### Data extraction and treatment

For LC-MS, raw datasets were extracted by the Molecular Feature Extraction tool in the MassHunter Qualitative Analysis software (Agilent Technologies). For GC-MS, deconvolution and data processing were performed with Automated Mass Spectrometry Deconvolution and Identification System (www.amdis.net). Due to retention time shifts during both LC−MS and GC-MS sequence runs, samples were multi-aligned using Mass Profiler Professional (Agilent Technologies). Subsequently, data filtration regarding quality assurance criteria (QA) and frequency in at least one of the compared groups (i.e., in 90% of samples) was applied.

### Statistical analysis and metabolite identification

Principal component analysis (PCA) was applied to check quality of the analysis, trends in the datasets and detect potential outliers. Then, multivariate statistics were used to select compounds with statistically significant differences between the groups. To select metabolites contributing the most into groups’ discrimination, orthogonal partial least squares discriminant analysis (OPLS-DA) was applied. Statistically significant metabolites were selected based on the Jack-knife confidence interval (*p*<0.05) and variable importance into projection (VIP) values. Compounds detected by LC-MS were first putatively identified using public available databases. The identity of compounds found in the databases was confirmed by LC−MS/MS analyses. For GC-MS, metabolites found to be statistically significant were identified based on comparison of their retention time, retention index and mass spectra with Fiehn RTL library, in-house target plasma library or NIST library.

A detailed description of analytical and statistical methods can be found in S1 File.

## Results

### Quality of the analysis

**Fig 1:**
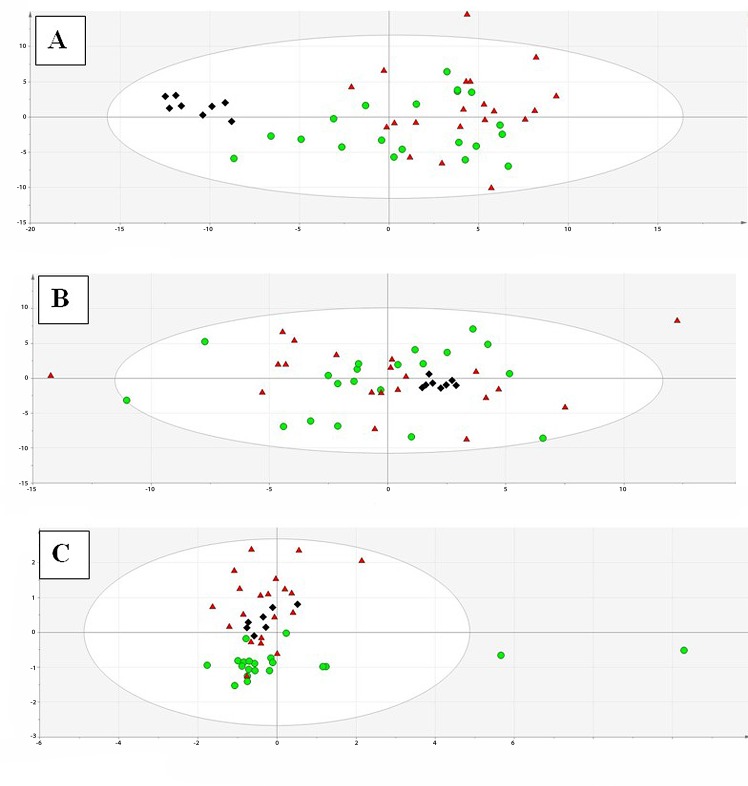
PCA models built with the data after filtration step. (A) Scores plot for a PCA model built with the data set obtained in LC−MS positive mode. Quality parameters for the model: explained variance R^2^ = 0.468, predicted variance Q^2^ = 0.227. (B) Scores plot for a PCA model built with the data set obtained in LC−MS negative mode. R^2^ = 0.427, Q^2^ = 0.139. (C) Scores plot for a PCA model built with the data set obtained in GC−MS. R^2^ = 0.567, Q^2^ = 0.193. QCs have been marked as black diamonds. Pulmonary hypertensive group (PAH) and control (C) have been marked as red triangles and green circles, respectively. PCA: principal component analysis; LC-MS: liquid chromatography-mass spectrometry; GC-MS: gas chromatography-mass spectrometry; QCs: quality control samples.

The obtained raw datasets, after alignment step, consisted of 27362, 12360 and 139 features for LC-ESI(+)-MS, LC-ESI(-)-MS and GC-MS, respectively. Data was initially filtered according to quality assurance criteria before performing PCA analysis. LC-MS, in positive and negative ionization modes, and GC-MS data matrices contained 1950, 1114 and 49 variables, respectively. QCs clustered adequately (Fig 1), confirming that the system was stable during analyses and that the differences between compared groups reflected true biological differences, and not artefacts derived from analytical variability. The Hottelling’s test was applied during PCA models’ construction to reveal potential outliers. In PCA model built on GC-MS dataset, one analytical outlier, which resulted to be a control sample, was detected and subsequently excluded from further statistical analysis of GC-MS data.

### Sample discrimination and metabolite identification

**Fig 2:**
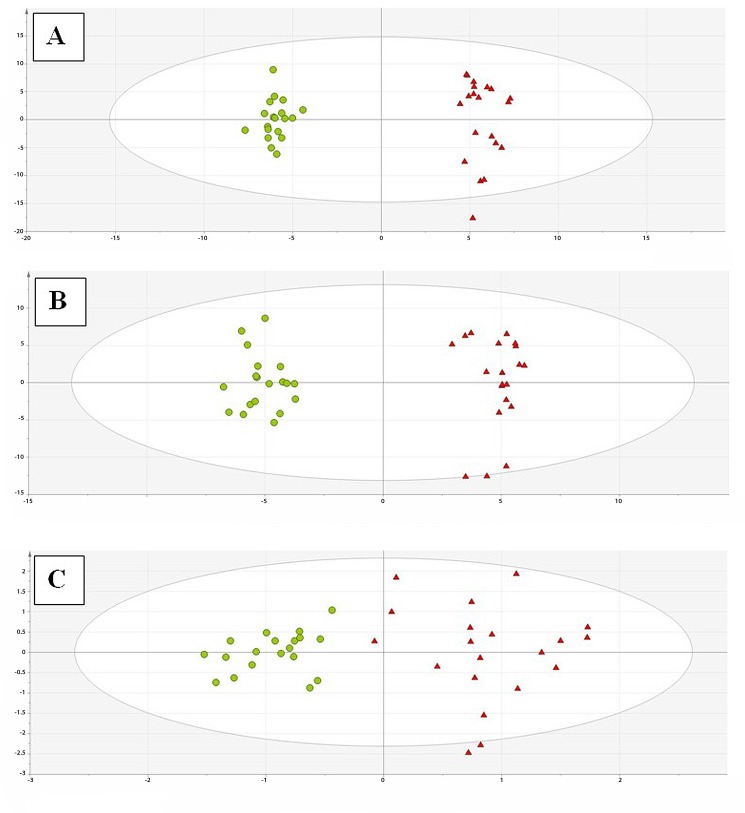
OPLS-DA plots for plasma metabolic fingerprints obtained from C and PAH groups. (A) OPLS-DA model (R^2^ = 0.844, Q^2^ = 0.653) for LC-MS data in positive ionization mode. (B) OPLS-DA model (R^2^ = 0.897, Q^2^ = 0.618) for LC-MS data in negative ionization mode. (C) OPLS-DA model (R^2^ = 0.825, Q^2^ = 0.649) for GC-MS data. Pulmonary hypertensive group (PAH) and control (C) have been marked as red triangles and green circles, respectively. OPLS-DA: orthogonal partial least squares discriminant analysis.

Before OPLS-DA, variables were further filtered based on minimum frequency in the study groups (required presence in at least 90% of samples in PAH or C group), 836, 637 and 40 variables remained for LC-MS in positive ionization mode, LC-MS in negative ionization mode, and GC-MS, respectively. OPLS-DA models (Fig 2) clearly separated the PAH and control groups in the 3 modalities. The quality of the OPLS-DA models was provided by explained variance R^2^ (0.982 for LC-MS positive ionization, 0.998 for LC-MS negative ionization, and 0.825 for GC−MS) and predicted variance Q^2^ (0.524 for LC-MS positive ionization, 0.502 for LC-MS negative ionization, and 0.649 for GC−MS). The results of metabolite identification that represented statistically significant differences between PAH and C group are presented in Tables 1 and 2. To select metabolites which contribute the most into groups’ discrimination, Jack-knife confidence intervals (*p*<0.05) and VIP values were checked (VIP >1). The compounds numbered 149, 63 and 9 were selected as statistically significant for LC-ESI(+)-MS, LC-ESI(-)-MS and GC-MS, respectively.

**Table 1:** Identified metabolites that significantly change in human plasma from PAH patients *vs*. Control group, detected in LC-MS.

Name	RT(min)	Ionization	Mono-isotopic mass	VIP	% Change *vs*. control	CV QCs [%]	Identification	*p* valueJK	FC
Nonanoic acid	12.7	positive	158.1304	1.0	28	9	159.1375, 141.9564,124.9622, 97.9679, 89.0596, 43.0545	0.0027	1.28
Eicosenoic acid	34.1	negative	310.2842	1.8	27	8	Database, isotopic distribution	0.0094	1.27
Azelaic acid	1.5	negative	188.1043	2.2	48	12	Database, isotopic distribution	0.0241	1.48
Octadecadienal	27.3	positive	264.2451	1.4	-22	9	Database, isotopic distribution	0.0113	0.78
Palmitoylcarnitine	18.1	positive	399.3347	1.5	31	5	400.3418, 341.2682, 85.0285, 60.0809	0.0027	1.31
Stearoylcarnitine	21.2	positive	427.3653	1.3	31	4	428.3725, 311.2927, 85.0281, 60.0799	0.0036	1.31
Dimethylnonanoylcarnitine	8.7	positive	329.2564	1.1	-21	10	329.2568, 271.1880, 85.0284, 60.0796	0.0114	0.79
Palmitamide	26.5	positive	255.2579	3.0	-18	7	256.2644, 102.0911, 116.1065, 88.0756, 57.0701	0.0154	0.82
Oleamide	27.3	positive	281.2741	1.2	-14	7	281.2735, 265.2529, 247.2420, 135.1166, 97.1010, 83.0856, 69.0701, 57.0702	0.0015	0.86
Stearamide	31.1	positive	283.2889	1.6	-21	12	284.2962, 116.1072, 102.0911, 88.0756, 57.0699	0.0031	0.79
Palmitoleamide	23.3	positive	253.2410	1.1	-25	8	Database, isotopic distribution	0.0081	0.75
Sphingosine	21.7	positive	311.2823	3.2	-42	12	Database, isotopic distribution	0.0214	0.58
Glycochenodeoxycholate sulphate	8.5	negative	529.2697	1.8	84	9	528.2620, 448.3069, 74.0236	0.0131	1.84
Glycochenodeoxycholate	11.7	negative	449.3134	1.2	66	11	Database, isotopic distribution	0.0065	1.66
Deoxycholic acid-3 glucuronide	8.1	negative	568.3228	1.5	63	9	567.3152, 505.3094, 391.2780, 113.0218, 85.0277, 75.0089, 44.9971	0.0244	1.63
Hydroxy-oxo-cholanoic acid	34.1	positive	390.2779	1.2	40	27	391.2852, 167.0361, 149.0225, 71.0851, 57.0703	0.0038	1.40
Glutamine	1.1	negative	146.0693	1.0	59	6	145.0615, 127.0500, 109.0396, 58.0316, 41.9997	0.0088	1.59
Tryptophan	1.1	positive	204.0897	2.5	-14	8	205.0984, 188.0706, 146.0599, 118.0651	0.0123	0.86
Uric acid	0.7	negative	168.0262	1.2	20	3	167.0195, 124.0147, 96.0198, 69.0089, 41.9990	0.0088	1.20
LPC	9.6	positive	511.3277	1.1	70	29	512.3277, 184.0726, 104.1070, 86.0963	0.0028	1.70
PC(26:1)	24.1	positive	647.4436	1.2	-29	26	648.4489, 184.0727, 104.1054, 86.0951	0.0034	0.71

Abbreviations: RT-retention time, VIP-variable importance to projection, CV QCs-coefficient of variation in QC samples, JK-Jack-knife confidence interval level, FC-fold-change

**Table 2:** Identified metabolites that significantly change in human plasma from PAH patients *vs*. Control group, detected in GC-Q-MS.

Name	T	Q	RT (min)	RI	VIP	% Change *vs*. control	CV QCs [%]	*p* value JK	FC
Lactic acid	117	191, 147, 73	6.7	733	1.4	123	10	0.0001	2.23
Pyruvic acid	174	89, 73, 59	6.6	721	1.0	71	19	0.0233	1.71
Threitol	73	147,217,103	12.9	1176	1.6	115	24	0.0065	2.15
Glycerol	205	147, 117, 73	9.8	945	0.9	175	14	0.0132	2.75
Cholesterol	129	329,73,368	27.6	2826	1.1	-9	27	0.0072	0.91
Urea	147	189, 73,66	9.4	923	1.0	39	17	0.0035	1.39
Creatinine	115	73,100,329	13.5	1232	1.3	77	29	0.0063	1.77
Aminomalonic acid	218	73,147,320	12.5	889	1.1	84	28	0.0113	1.84
Isoleucine/Norleucine	86	75,69, 188	8.4	853	1.5	-21	24	0.0022	0.79

Abbreviations: T-target ion, Q-qualifier ions, RI-retention index, CV QCs-coefficient of variation in QC samples, JK-Jack-knife confidence interval level, FC-fold-change

To avoid over-fitting and check the predictive value of the obtained OPLS-DA models, the leave-one-third-out cross-validation method was applied with the use of Matlab 2013b software. The percentages of samples that were correctly classified by each OPLS-DA model were calculated as the average of all prediction steps, which were 83% ± 12%, 92% ± 8%, and 83% ± 14% for LC−MS positive mode, LC−MS negative mode, and GC−MS, respectively. The root mean squares errors of cross-validation (RMSECV) were 0.3233, 0.2873 and 0.3277 for LC−MS positive mode, LC−MS negative mode, and GC−MS, respectively.

Each metabolite was externally validated by univariate statistical comparisons in an independent case and control study using multiple correction methods and performed in the validation study (Table 3).

**Table 3:** Results of univariate statistical analysis in the independent validation study.

Metabolite	*p*-value	*p*-value FDR
Lactic acid	0.001	0.010
Pyruvic acid	0.096	0.108
Threitol	0.001	0.016
Glycerol	0.035	0.044
Cholesterol	0.003	0.017
Urea	0.011	0.033
Creatinine	0.016	0.024
Aminomalonic acid	0.011	0.024
Isoleucine/norleucine	0.953	0.953
Nonanoic acid	0.998	0.998
Eicosenoic acid	0.674	0.787
Azelaic acid	0.889	0.889
Octadecadienal	0.137	0.187
Palmitoylcarnitine	0.023	0.031
Stearoylcarnitine	0.027	0.039
Palmitamide	0.029	0.047
Oleamide	0.021	0.041
Stearamide	0.020	0.044
Palmitoleamide	0.127	0.188
Sphingosine	0.046	0.093
Glycochenodeoxycholate sulphate	0.029	0.036
Glycochenodeoxycholate	0.370	0.648
Deoxycholic acid-3 glucuronide	0.034	0.039
Hydroxy-oxo-cholanoic acid	0.309	0.332
Glutamine	0.032	0.036
Tryptophan	0.024	0.036
Uric acid	0.626	0.878
LPC	0.283	0.330
PC(26:1)	0.179	0.228

Abbreviations: FDR- False Discovery Rate

## Discussion

In this study we identify, for the first time, systemic metabolic differences in plasma in PAH patients with respect to controls using a multimodality metabolomics approach. The metabolites identified are associated with various metabolic pathways including glycolysis, pentose phosphate pathway (PPP), lipid and fatty acid metabolism, amino acid metabolism, tricarboxilic acid (TCA) cycle and urea cycle. Interestingly, we found other metabolites not previously associated with PAH (i.e., threitol, aminomalonic acid) that may explain particular features of PAH and also could be used as biomarkers of the disease.

Lactate was markedly elevated in PAH patients, suggesting a shift in glucose metabolism towards glycolysis for ATP production at the expense of oxidative phosphorylation, the “so-called” Warburg effect. This shift provides a rapid route for ATP generation and prevents excessive mitochondrial reactive oxygen species production [8]. Although this metabolic switch is well established in rapidly proliferating endothelial and smooth muscle cells during the vascular remodelling of pulmonary hypertension [9], a recent report by Zhao’s et al. has shown reduced glycolysis in human PAH lung compared to normal tissue [7]. This discrepancy might be explained because Zhao’s et al. used lung samples with severe PAH rather than samples of patients with less severe PAH, which is the case of previous reports and our own study. Also, the analysis of lung biopsies (local metabolome) instead of blood plasma (whole-body metabolome) may partly explain this difference. Other metabolites related to carbohydrate metabolism, predominantly threitol, were increased in the PAH group. Threitol is an end product of xylose metabolism that is connected to the PPP through the glucuronate pathway [10]. The levels of threitol and other polyols are altered in diseases associated with carbohydrate metabolism derangements such as diabetes mellitus [11]. Together, these results suggest a deregulated carbohydrate metabolism in PAH patients, where the intermediates produced by the high glycolytic rate could feed the PPP for the production of nucleotides, amino acids and nicotinamide adenine dinucleotide phosphate (NADPH) needed to support the increased biosynthetic demands during cell proliferation, similarly to cancer [12].

The disrupted energy production is also connected to alterations in lipid and fatty acid metabolism. We found increased levels of free fatty acids, in particular nonanoic acid, and glycerol in the plasma of PAH patients, suggesting enhanced lipolysis. During starvation/exercise, glycerol and free fatty acids are released from adipose tissue (lipolysis) for energy generation. Free fatty acids are used as respiratory fuel by the tissues and glycerol is taken into hepatocytes for gluconeogenesis. In a healthy situation, insulin prevents lipolysis and inhibits hepatic gluconeogenesis, but not in diabetes or in the metabolic syndrome [13]. In addition, we observed a significant increase of long-chain fatty acylcarnitines in the PAH group. Long-chain acylcarnitines facilitate the transfer of long-chain fatty acids from cytoplasm into mitochondria during fatty acid β-oxidation (FAO). Acylcarnitines accumulate and are released into the circulation due to FAO defects [14] and can also be reflective of incomplete long-chain FAO, as it occurs in type 2 diabetes and in insulin resistance [15]. The accumulation of even long-chain acylcarnitines of 18-carbons (stearoylcarnitine) and 16-carbons (palmitoylcarnitine) found here points to an incomplete FAO, which might be associated with the impairment of the TCA cycle known to occur in PAH [16]. Also, the alterations in the fatty acid metabolism might be associated in part to an increase in the fatty acid ω-oxidation, as it has been shown to occur in lungs from advanced PAH patients [7]. Activation of this pathway could help to alleviate the failure of the fatty acid β-oxidation. Thus, as a consequence of the metabolic switch to cytoplasmic glycolysis, there is a marked reduction in the metabolism of pyruvate into the mitochondria, causing decreased energy production from oxidative phosphorylation and TCA cycle. The reduction in TCA cycle capacity is likely to contribute to a more inefficient long-chain FAO. Interestingly, it is becoming increasingly recognized that PAH patients exhibit features of the metabolic syndrome and insulin resistance [17]. This could explain, at least in part, some of the alterations in lipid and fatty acid metabolism and the deregulated carbohydrate metabolism observed in PAH. Furthermore, these metabolic alterations might be related to the hypoxemic state of the disease. Accordingly, our clinical data show mild arterial hypoxemia (PaO_2_ < 80 mmHg, see S1 and S2 Tables) in the PAH group, most likely due to the abnormal pulmonary vascular remodelling that occurs in PAH. Under reduced oxygen availability, there is increased expression of the hypoxia-inducible factor (HIF-1) in the affected tissues, as is the case of human pulmonary artery endothelial [18] and smooth muscle cells [19]. As the master regulator of the hypoxic response, HIF-1 up-regulates an array of enzymes that activate anaerobic glycolysis and suppress the oxidative phosphorylation and TCA cycle, playing a predominant role in PAH induction [8].

In addition to energy stores and structural components of cell membranes, lipids are important components of the endocrine system producing bioactive substances that influence metabolic and vascular homeostasis. We found a significant decrease in the levels of palmitamide, stearamide and oleamide in the plasma of patients with PAH. These compounds are fatty acid amides (FAAs), a large class of endogenous signalling lipids of growing interest. Through interactions with cannabinoid, vanilloid and peroxisome proliferator-activated receptors (PPARs), FAAs regulate a variety of pathophysiological functions such as appetite, lipid and glucose metabolism, vasodilation, cardiac function and inflammation [20]. Many actions of FAAs are potentially protective to the cardiovascular system. For instance, FAAs are potent regional vasodilators [21]. FAAs also play a role in the regulation of energy metabolism by stimulating lipolysis and fatty acid oxidation [22], and have potential anti-diabetic effects [23]. These observations suggest that down-regulation of FAAs might contribute to some of the metabolic derangements found in PAH.

Similarly, we found a significant decrease of sphingosine in the plasma of PAH patients. Sphingosine is a potent bioactive signalling lipid involved in the regulation of processes such as cell growth, differentiation, and apoptosis [24]. Like FAAs, sphingolipids have been suggested to be agonists of PPARs [25], and therefore, alterations in the levels of sphingolipids might have an impact on lipid metabolism, fatty acid oxidation and glucose homeostasis [26]. Of note, a recent study has shown significant elevation of sphingosine-1-phosphate (S1P) in lung tissues from patients with severe PAH [27]. S1P elevation was accompanied by increased expression of sphingosine kinase 1 (SphK1), the predominant isoform responsible for the synthesis of S1P from sphingosine in the lungs. Thus, the reduction of sphingosine found in our study might be consequence of an increase in SphK1 and hence in the production of S1P, causing depletion of the substrate for S1P production (sphingosine). This interpretation will need to be corroborated in future targeted studies covering different stages of disease severity.

Our study also demonstrates changes in cholesterol metabolism in PAH. While there was a slight reduction in free cholesterol, we observed a substantial elevation of cholesterol-derived bile acids and some of their metabolites (glycochenodeoxycholate sulphate and deoxycholic acid 3-glucuronide). Besides facilitating the absorption of dietary lipids and fat-soluble vitamins, bile acids regulate glucose homeostasis, lipid and lipoprotein metabolism, energy expenditure, and inflammation through interaction with nuclear receptors (farnesoid X, pregnane X, vitamin D receptors) and with GPBAR1 (G protein-coupled bile acid receptor 1) [28]. Importantly, a recent study has shown elevation of bile acid metabolites in lung tissue from PAH patients; the authors suggested that the *de novo* biosynthesis of bile acids was primarily localized in pulmonary vascular endothelial cells [29]. These findings suggest a potential role for bile acids in PAH pathogenesis and the need for further studies to evaluate this family of metabolites as biomarkers of disease progression.

Other metabolites with significant changes in PAH comprise amino acids. Noteworthy, we detected a substantial increase in circulating glutamine in the PAH group (59% *vs*. control). Glutamine is the most abundant free amino acid in plasma and is utilized at high rates by rapidly dividing cells (glutaminolysis) [30]. Glutamine can be hydrolysed to glutamate and subsequently converted to α-ketoglutarate in the mitochondria. α-ketoglutarate can enter the TCA cycle and replenish the metabolic intermediates (anaplerosis), thereby supporting the biosynthetic demands for NADPH and fatty acids during rapid cell growth [30], as in cancer [12]. Importantly, a recent study has shown increased levels of plasmatic glutamine in a model of monocrotaline-induced PAH [31].

On the other hand, tryptophan was decreased in the PAH group, which can be of relevance for PAH pathogenesis. Tryptophan is converted to serotonin by the enzyme tryptophan hydroxylase (coded in the gene TPH1). Serotonin has been suggested to enhance pulmonary arterial smooth muscle cell proliferation, vasoconstriction and microthrombosis [32]. TPH1 is mainly expressed in the gut and mediates the generation of serotonin in the periphery [33]. Interestingly, increased TPH1 expression has been reported in lungs and in pulmonary endothelial cells from patients with idiopathic PAH [34]. Moreover, a study aimed to investigate the effect of TPH1 deficiency on hypoxia-induced PAH in mice confirmed its crucial role in disease development [35]. In our study, the decrease in plasma tryptophan might be indicative of its metabolic conversion into serotonin, which could enhance cell proliferation and vascular remodelling in PAH. However, this suggestion needs to be confirmed in further studies assessing the ratio tryptophan/serotonin and possible correlation with disease progression.

**Fig 3:**
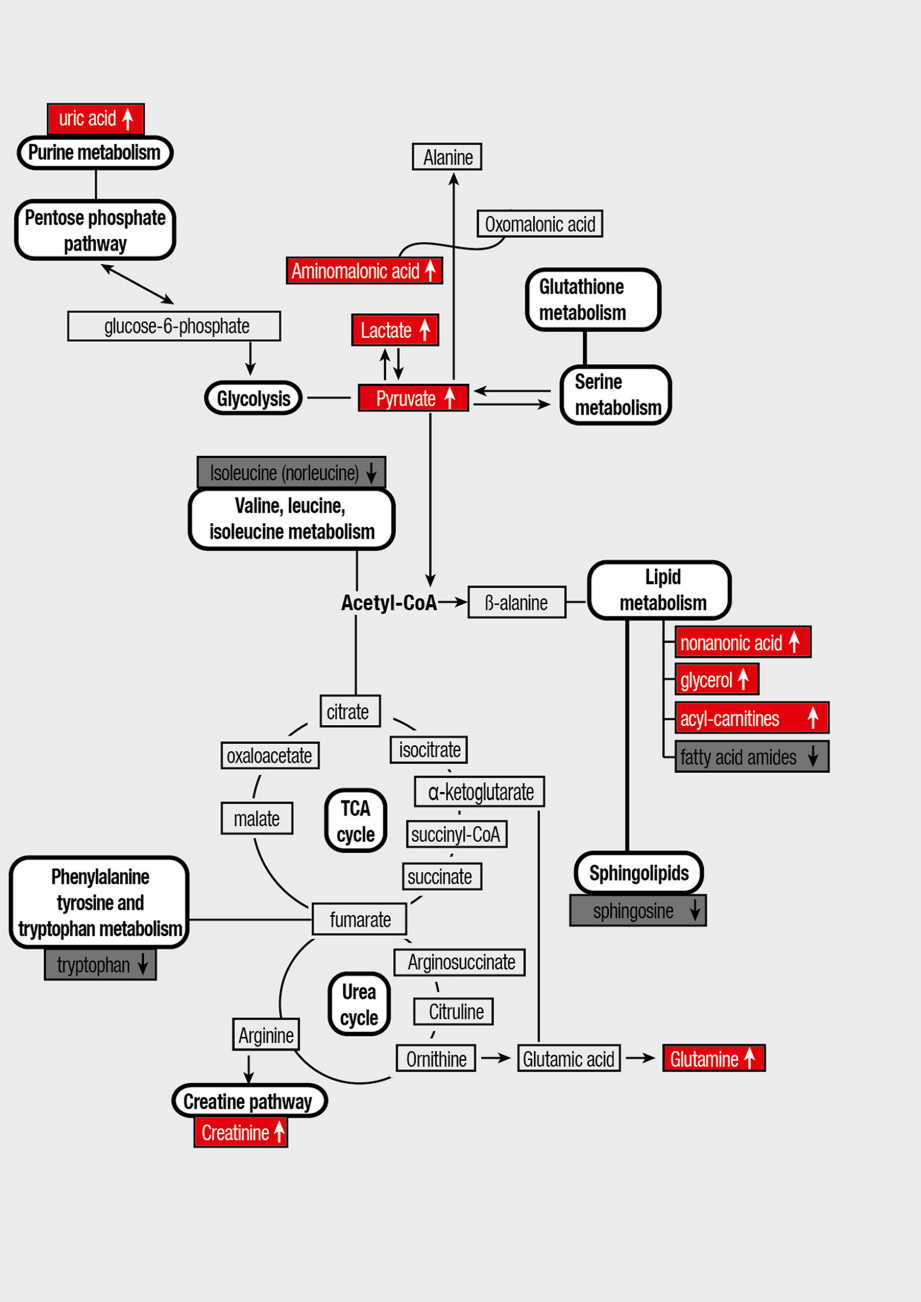
The connections between metabolic pathways altered in plasma of PAH patients as compared to control group. TCA: tricarboxylic acid.

Interestingly, a substantial increase of aminomalonic acid was detected in the PAH group. Although the origin of this metabolite in blood has not been well elucidated yet, aminomalonic acid can be routinely detected in the serum metabolome of healthy individuals by GC-MS [36]. Aminomalonic was first identified in human atherosclerotic plaques [37], and later was associated with the presence of foam cells and macrophage-rich tissues in atherosclerotic rabbits (aorta, blood, spleen, lungs) [38]. Besides, the origin of aminomalonic acid has been associated with free-radical mediated oxidation of amino acid residues in proteins, mainly glycine and cysteine [39]. Recently, increased plasma level of aminomalonic acid was observed in patients with large aneurysms [40], suggesting a role for aminomalonic acid in cardiovascular diseases with an inflammatory and/or oxidative stress component. Consistent with this, our clinical data showed elevated levels of the inflammatory marker C-reactive protein in the PAH group (PAH 0.65±0.73 *vs*. Control 0.22±0.28, *p*<0.001, see S1 and S2 Tables). Since increasing evidence indicates that inflammation and oxidative stress play an important role in the pathophysiology of PAH [41], aminomalonic acid emerges as a potential biomarker regarding the inflammatory/oxidative extent. The connections between metabolic pathways altered in PAH patients are presented in Fig 3 and in S2 File.

This study has some limitations, the most important one, the sample size. However, as the first study applying this type of methodology, the reference values provided here can help sizing future studies to explore particular aspects of the disease. On the other hand, due to the extreme rarity of PAH in humans, accruing a large sample is difficult, time-consuming and requires multicenter studies. Due to the small sample size, we could not stratify by time from diagnosis or background therapy. Further studies on larger samples that can profit from the use of quantitative targeted multiplatform metabolomics will be able to confirm the potential markers identified in this study. Additionally, a joint analysis of the proposed metabolic markers together with clinical and hemodynamic parameters in PAH patients can provide further advancements in clinical and diagnostic practices.

In summary, in this study, plasma untargeted multiplatform metabolomics followed by bioinformatics data analysis provides data on diagnostic markers and pathophysiological mechanisms occurring in PAH. Metabolites significantly changed in PAH patients as compared to control were associated with glycolytic shift, lipid-related energy imbalance as well as cellular signal transduction. These metabolic abnormalities could be considered as pathological hallmarks of PAH. Finally, plasma metabolome includes the biochemical information from all organs of the body. Based on clinical characteristics of participants (S1 and S2 Tables), there are no clear symptoms and analytical changes that can be attributed to other different pathologies. However, we cannot completely exclude that PAH affects in a very late stage the metabolism of different organs from lung to heart. Further metabolic studies on specific organs, such as liver or kidney, would clarify this concern.

## Supporting Information

S1 File A detailed description of materials and methods applied in the study. (PDF)

S2 File Metabolite Set Enrichment Analysis. (PDF)

S1 Table Study cohort. Clinical characteristics of participants.
(PDF)

S2 Table Validation cohort. Clinical characteristics of participants.
(PDF)
