# Supplementary material for: New Biochemical Insights into the Mechanisms of Pulmonary Arterial Hypertension in Humans
Source: PLoS One. 2016 Aug 3;11(8):e0160505. doi: 10.1371/journal.pone.0160505 (PMC4972307; doi:10.1371/journal.pone.0160505)
Supplement: S1 File — (PDF) [file pone.0160505.s001.pdf]

NEW BIOCHEMICAL INSIGHTS INTO THE MECHANISMS OF PULMONARY ARTERIAL HYPERTENSION IN HUMANS

Renata Bujak, Jesús Mateo, Isabel Blanco, Danuta Dudzik, Michał J. Markuszewski, Victor Ivo Peinado, Martín Laclaustra, Joan Albert Barberá, Coral Barbas, Jesús Ruiz-Cabello

SUPPORTING INFORMATION

Material and Methods

Study design and samples

This case-control study included 20 patients with confirmed PAH (PAH group) derived from Clinic University Hospital in Barcelona and 20 healthy controls. Independent recruitment of additional patients (n=20) and controls (n=12) that were processed in a separated batch and not used in the main analyses allowed external validation. Fasting blood samples were drawn and plasma was frozen for metabolic fingerprinting. Metabolomics included Liquid Chromatography-Mass Spectrometry (LC-MS) in positive and negative modes and Gas Chromatography-Mass Spectrometry (GC-MS), which conveyed a series of processes including deproteinization and metabolite extraction, pooling of samples to prepare quality control samples, data extraction and deconvolution (adducts detection and peak alignment), and filtering according to quality assurance criteria and to a high (90%) shared presence in at least one of the comparison groups.

Plasma metabolic fingerprinting with HPLC-ESI-QTOF-MS

Plasma samples, before LC-MS analysis, were prepared using the procedure previously reported [1]. Deproteinization and metabolite extraction were performed by adding 50 µl of plasma to 150 µL of a cold (−20°C) mixture of methanol and ethanol (1:1 *v/v*). Samples were then vortexed for 1 min, stored for 5 min at -20°C, and vortexed again for a few seconds. The pellet was removed by centrifuging at 15700x*g* for 20 min at 4°C, and the obtained supernatant was filtered through a 0.22 µm nylon filter. Together with plasma samples, eight quality control samples (QCs) were prepared as aliquots of a pool of equal volumes from all samples included in the study. QCs were treated with the same preparation procedure as the rest of the samples.

Samples were analysed by a HPLC system (1200 Infinity series, Agilent Technologies, Waldbronn, Germany) consisting of a degasser, two binary pumps, and thermostated auto-sampler connected to an Agilent Technologies QTOF (6520) mass

spectrometry detector. Electrospray ionization (ESI) was used as an ion source. Extracted plasma samples (10 μL) were injected onto a reversed-phase column (Discovery HS C18, 15 cm x 2.1 mm, 3 μm; Supelco) with a guard column (Discovery HS C18; 2 cm x 2.1 mm, 3 mm; Supelco) thermostated at 40°C. The system was operated in positive and negative modes at 0.6 mL/min flow rate with solvent A, water with 0.1% formic acid, and solvent B, acetonitrile with 0.1% formic acid. Gradient started from 25% B to 95% B in 35 min, and returned to starting conditions in 1 min, keeping the re-equilibration at 25% B for 9 min. The detector operated in full scan mode, from 50 to 1000 m/z for positive mode and from 50 to 1100 m/z for negative mode, with a scan rate of 1 scan per second. Accurate mass measurements were obtained by means of an automated calibrant delivery system using dual ESI source that continuously introduced a calibration solution, with reference masses at m/z 121.0509 (protonated purine) and m/z 922.0098 [protonated hexakis(1H,1H,3Htetrafluoropropoxy) phosphazine or HP-921] in positive ion mode; and m/z 112.9856 (TFA anion) and m/z 1033.9881 [hexakis(1H,1H,3H-tetrafluoropropoxy)phosphazine or HP-0921] in negative ion mode. The capillary voltage was set to 3000 V for positive and 4000 V for negative ionization mode, and the nebulizer gas flow rate was 10.5 L/min. Randomized samples were analysed in two separate runs (first for positive and second for negative mode). QC samples were analysed at the beginning of the each sequence run to equilibrate column and system, and then after every 5 plasma samples to control system stability and reproducibility.

Plasma metabolic fingerprinting with GC-EI-Q-MS

Plasma samples were prepared for GC−MS analysis applying previously reported procedure [2]. First steps of plasma sample preparation were: deprotenization with acetonitrile (1:3 *v/v*) and centrifugation (15400x*g*, 20 min, 4°C). After the protein precipitation, the obtained supernatant was transferred to another vial with glass insert and then evaporated to dryness in a Speedvac Concentrator (Thermo Fisher Scientific, Waltham, MA, USA). Derivatization was performed using two-step procedure. The methoxymation step with O-methoxyamine hydrochloride in pyridine was carried out overnight. BSTFA with 1% TMCS was then added, and after silylation (1h, 70°C), each sample was re-dissolved in heptane with C18:0 methyl ester (IS). GC−MS analysis was performed by a 7890A gas chromatography instrument (Agilent Technologies, Santa Clara, CA, USA) interfaced to inert MSD with Quadrupole (Agilent Technologies 5975). 2 μL of derivatised plasma samples was injected in split mode using an Agilent Technologies 7693 auto-sampler onto a GC column DB5-MS (30

m length, 0.25 mm i.d., 0.25 μm film 95% dimethyl/5% diphenylpolysiloxane) with an integrated pre-column (10 mJ&W) from Agilent Technologies. Carrier gas (He) flow rate was set at 1 mL/min and injector temperature at 250°C. Split ratio was fixed from 1:5 to 1:10 with 3 to 10 mL/min He split flow into a Restek 20782 (Bellefonte, PA, USA) deactivated glass-wool split liner. Temperature gradient was programmed: initial oven temperature was set at 60°C (held for 1 min), then increased to 325°C at 10°C/min, and finally a cool-down period was applied for 10 min before the next injection. Total time of analysis was 37.5 min. Detector transfer line, filament source, and quadrupole temperatures were set at 290°C, 230°C, and 150°C, respectively. Voltage for electron impact (EI) ionization source was 70 eV. The mass spectrometer was operated in scan mode over a mass range of 50−600 m/z at 2 spectra/s. Similar to LC-MS analysis, QCs were prepared in the same time and with the same procedure as plasma samples included in the study. Regular injection of QC samples (every 5 plasma samples) was performed during the randomized sequence run. At the beginning of the GC-MS analysis, the mixture of *n*-alkanes (C8−C28) dissolved in *n*-hexane was run for retention index calculations. Data acquisition, peak detection and spectra processing were performed with ChemStation E.02.00.493 software (Agilent Technologies).

Data extraction and treatment

Since co-elution is a common phenomenon in MS-based metabolomics, data extraction and deconvolution are first steps in data treatment procedure. In case of LC-MS, raw datasets were cleaned of background noise and unrelated ions by the Molecular Feature Extraction (MFE) tool in the MassHunter Qualitative Analysis B.06.00 software (Agilent Technologies). The MFE algorithm groups ions regarding charge state, isotopic distribution, and/or the presence of adducts and dimers by using the accuracy of the mass measurements. After MFE processing, each compound is characterized by mass, retention time, and abundance. Parameters selected for data extraction were similar to previously described [3,4]. The background noise limit was set to 200 counts, and to find co-eluting adducts of the same feature, the following adduct settings were applied: +H, +Na, +K and neutral loss of water in positive ionization, and: −H, +HCOO and neutral loss of water for negative ionization. Due to retention time shifts during LC−MS sequence run, peak alignment is mandatory to provide that the same compound (potential metabolite) is marked as the same entity in all analysed samples. Therefore, Samples were multi-aligned using Mass Profiler Professional (B.12.01, Agilent Technologies). Alignment parameters were 1.0% for retention time correction and 20 ppm for mass correction. Then, the obtained datasets in positive and negative ionization

modes were filtered according to quality assurance (QA) criteria [5]. Compounds that were present in at least 50% of QC samples and their coefficient of variation (CV) were lower than 30% were considered for further data treatment and statistical analysis. Then, data matrices were filtered by selecting only the compounds that were present in at least 90% samples in at least one of the studied groups (i.e., in 90% samples of C group or in 90% samples of PAH group). The obtained datasets were used for further statistical analysis.

In the case of GC-MS data, total ion chromatograms (TICs) were checked regarding quality of chromatograms and internal standard signal. Deconvolution and data processing were automatically performed with Automated Mass Spectrometry Deconvolution and Identification System (AMDIS, www.amdis.net). Identification of the deconvoluted compounds was based on retention time (RT), retention index (RI), and mass spectrum. RI for each metabolite was calculated by normalization of its RT by the RT and RI of the closest eluting n-alkane, present in the mixture of fatty acid methyl esters, which was analysed at the beginning of the GC-MS sequence run. Metabolites detected in plasma extracts were identified based on comparison of their mass spectrum and RI with those in the Fiehn RTL library. Moreover, mass spectra of compounds not found in the Fiehn RTL library, were searched using the NIST mass spectral library. Multi-alignment was carried out with the use of Mass Profiler Professional B.12.01 software (Agilent Technologies). Subsequently, data filtration regarding QA criteria (the same as in the case of LC-MS data) and frequency in at least one of compared group (i.e., in 90% samples of C group or in 90% samples of PAH group) were applied. Before statistical analysis, data matrix was normalized by the internal standard.

Statistical analysis and metabolite identification

Multivariable statistics were used to select compounds that represented statistically significant differences between the PAH and C groups. First, principal component analysis (PCA) was applied to reveal general trends in the dataset, check quality of the analysis and detect potential outliers. PCA is an unsupervised method and no information about group membership was used during the multivariable modelling. To build PCA models for independent datasets obtained with LC-MS (in positive and negative ionization modes) and GC-MS, data matrices consisted of variables that passed QA filtering and samples frequency filtering criteria (see section 2.4). However, to select variables more closely related to group classification and consequently correlated with the pathological condition in PAH state, a supervised method, orthogonal partial least squares discriminant analysis (OPLS-DA) was

applied in the study. To build OPLS-DA models, variables were further filtered based on presence (90% of all samples) in at least one of the groups. To select metabolites which contributed the most into groups' discrimination, the Jack-knife confidence interval ($p<0.05$) and variable importance into projection (VIP) values were checked after creation of OPLS-DA models. Statistically significant compounds, detected with the use of LC-MS technique, were first putatively identified by searching for public available databases: METLIN (www.metlin.scripps.edu), KEGG (www.genome.jp/kegg), and LIPIDMAPS (www.lipidmaps.org/), all simultaneously accessed by the recently introduced search engine, CEU MassMediator (http://ceumass.eps.uspceu.es/mediator). For complementary information HMDB (www.hmdb.ca) was also used. The identity of compounds found in the databases mentioned above was confirmed by LC−MS/MS by using the same QTOF instrument. Experiments were conducted with the same chromatographic conditions as first untargeted analysis. Selected ions were targeted for collision induced dissociation (CID) fragmentation on the fly based on the previously determined accurate mass and retention time.

In case of GC-MS, the metabolites that were statistically different in the compared groups were identified based on comparison of their RT, RI and mass spectra with those available either in Fiehn RTL library, in-house target plasma library or NIST library.

The external validation of potential markers selected by multivariate statistics in the first study (PAH *vs.* C) was performed in an independent validation set (see section 2.1). Plasma metabolic fingerprinting experiment was performed using the same analytical instruments and methods as described in sections 2.2. and 2.3. To select compounds that represented statistically significant differences ($p<0.05$) univariate statistical analysis was performed in Matlab 2013b software. The normality of the data distribution was checked using Shapiro-Wilka test. For normally distributed variables, the parametric standard or Welch's unpaired *t*-test (according to the F-test for homogeneity of variances) were used. In the case of not normally distributed variables, the nonparametric Mann-Whitney *U*-test was applied. The multiple correction of calculated *p*-values was performed with the use of Benjamini-Hochberg False Discovery Rate (FDR) method.

Online Supplementary References

[1] Ciborowski M, Teul J, Martin-Ventura JL, Egido J, Barbas C. Metabolomics with LC-QTOF-MS permits the prediction of disease stage in aortic abdominal aneurysm based on plasma metabolic fingerprint. PLoS One 2012; 7: e31982. doi: 10.1371/journal.pone.0031982.

[2] Garcia A, Barbas C. Gas Chromatography-Mass Spectrometry (GC-MS)-based metabolomics. In Metabolic Profiling, Methods in Molecular Biology; Metz, T. O., Ed.; Springer: New York, NY, 2011; Vol. 708, pp 191−204.

[3] Whiley L, Godzien J, Ruperez FJ, Legido-Quigley C, Barbas C. In-vial dual extraction for direct LC-MS analysis of plasma for comprehensive and highly reproducible metabolic fingerprinting. Anal Chem 2012; 84: 5992−5999.

[4] Ciborowski M, Lipska A, Godzien J, Ferrarini A, Korsak J, Radziwon P, Tomasiak M, Barbas C. Combination of LC−MS- and GC−MS-based metabolomics to study the effect of ozonated autohemotherapy on human blood. J Proteome Res 2012; 11: 6231−6241.

[5] Dunn WB, Broadhurst D, Begley P, Zelena E, Francis-McIntyre S, Anderson N, BrownM, Knowles JD, Halsall A, Haselden JN, Nicholls AW, Wilson ID, Kell DB, Goodacre R. The Human Serum Metabolome (HUSERMET) Consortium. Procedures for large-scale metabolic profiling of serum and plasma using gas chromatography and liquid chromatography coupled to mass spectrometry. Nat Protoc 2011; 6: 1060-1083.
