# Supplementary material for: New Biochemical Insights into the Mechanisms of Pulmonary Arterial Hypertension in Humans
Source: PLoS One. 2016 Aug 3;11(8):e0160505. doi: 10.1371/journal.pone.0160505 (PMC4972307; doi:10.1371/journal.pone.0160505)
Supplement: S2 File — (PDF) [file pone.0160505.s002.pdf]

Metabolomic Data Analysis with MetaboAnalyst 3.0

User ID: guest4338426544980214902

June 3, 2016

1 Background

MESA or Metabolite Set Enrichment Analysis is a way to identify biologically meaningful patterns that are significantly enriched in quantitative metabolomic data. In conventional approaches, metabolites are evaluated individually for their significance under conditions of study. Those compounds that have passed certain sigificance level are then combined to see if any meaningful patterns can be discerned. In contrast, MSEA directly investigates if a set of functionally related metabolites without the need to preselect compounds based on some arbituary cut-off threshold. It has the potentail to identify subtle but consistent changes among a group of related compounds, which may go undetected with the conventional approaches.

Essentially, MSEA is a metabolomic version of the popular GSEA (Gene Set Enrichment Analysis) software with its own collection of metabolite set libraries as well as an implementation of user-friendly web-interfaces. GSEA is widely used in genomics data analysis and has proven to be a powerful alternative to conventional approaches. For more information, please refer to the original paper by Subramanian A, and a nice review paper by Nam D, Kim SY.

2 MSEA Overview

Metabolite set enrichment analysis consists of four steps - data input, data processing, data analysis, and results download. Different analysis procedures are performed based on different input types. In addition, users can also browse and search the metabolite set libraries as well as upload their self-defined metabolite sets for enrichment analysis. Users can also perform metabolite name mapping between a variety of compound names, synonyms, and major database identifiers.

3 Data Input

There are three enrichment analysis algorithms offered by MSEA. Accordingly, three different types of data inputs are required by these three approaches:

- A list of important compound names - entered as a one column data (*Over Representation Analysis (ORA)*);
- A single measured biofluid (urine, blood, CSF) sample- entered as tab separated two-column data with the first column for compound name, and the second for concentration values (*Single Sample Profiling (SSP)*);
- A compound concentration table - entered as a comma separated (.csv) file with the each sample per row and each metabolite concentration per column. The first column is sample names and the second column for sample phenotype labels (*Quantitative Enrichment Analysis (QEA)*)

You selected Over Representation Analysis (ORA) which requires a list of compound names as input.

4 Data Process

The first step is to standardize the compound labels. It is an essential step since the compound labels will be subsequently compared with compounds contained in the metabolite set library. MSEA has a built-in tool to convert between compound common names, synonyms, identifiers used in HMDB ID, PubChem, ChEBI, BiGG, METLIN, KEGG, or Reactome. **Table 1** shows the conversion results. Note: *1* indicates exact match, *2* indicates approximate match, and *0* indicates no match. A text file contain the result can be found the downloaded file *name_map.csv*

Table 1: Result from Compound Name Mapping

	Query	Match	HMDB	PubChem	KEGG	Comment
1	Lactic acid	L-Lactic acid	HMDB00190	107689	C00186	1
2	Pyruvic acid	Pyruvic acid	HMDB00243	1060	C00022	1
3	Threitol	D-Threitol	HMDB04136	169019	C16884	1
4	Glycerol	Glycerol	HMDB00131	753	C00116	1
5	Cholesterol	Cholesterol	HMDB00067	11025495	C00187	1
6	Urea	Urea	HMDB00294	1176	C00086	1
7	Creatinine	Creatinine	HMDB00562	588	C00791	1
8	Aminomalonic acid	Aminomalonic acid	HMDB01147	100714	C00872	1
9	Isoleucine	(+/-)-erythro-Isoleucine	HMDB33923	791	C16434	1
10	Nonanoic acid	Pelargonic acid	HMDB00847	8158	C01601	1
11	Eicosenoic acid	Eicosenoic acid	HMDB02231	5282768	C16526	1
12	Azelaic acid	Azelaic acid	HMDB00784	2266	C08261	1
13	Octadecadienal	NA	NA	NA	NA	0
14	Palmitoylcarnitine	NA	NA	NA	NA	0
15	Stearoylcarnitine	Stearoylcarnitine	HMDB00848	6426855		1
16	Palmitamide	Palmitic amide	HMDB12273	69421		1
17	Oleamide	Oleamide	HMDB02117	5283387	C19670	1
18	Stearamide	Octadecanamide	HMDB34146	31292	C13846	1
19	Palmitoleamide	NA	NA	NA	NA	0
20	Sphingosine	Sphingosine	HMDB00252	5353955	C00319	1
21	Glycochenodeoxycholate sulphate	NA	NA	NA	NA	0
22	Glycochenodeoxycholate	Chenodeoxycholic acid glycine conjugate	HMDB00637	22833540	C05466	1
23	Deoxycholic acid-3 glucuronide	Deoxycholic acid 3-glucuronide	HMDB04903	22833627	C06136	1
24	Hydroxy-oxo-cholanoic acid	NA	NA	NA	NA	0
25	Glutamine	L-Glutamine	HMDB00641	5961	C00064	1
26	Tryptophan	D-Tryptophan	HMDB13609	9060	C00525	1
27	Uric acid	Uric acid	HMDB00289	1175	C00366	1

The second step is to check concentration values. For SSP analysis, the concentration must be measured in *umol* for blood and CSF samples. The urinary concentrations must be first converted to *umol/mmol_creatinine* in order to compare with reported concentrations in literature. No missing or negative values are allowed in SSP analysis. The concentration data for QEA analysis is more flexible. Users can upload either the original concentration data or normalized data. Missing or negative values are allowed (coded as *NA*) for QEA. Please note, MSEA does not perform data normalization. If normalization is important, you should first normalize your data before upload. You can use our companion website **MetaboAnalyst** *www.metaboanalyst.ca* for a variety of data processing and normalization methods.

5 Selection of Metabolite Set Library

Before proceeding to enrichment analysis, a metabolite set library has to be chosen. There are seven built-in libraries offered by MSEA:

- Metabolic pathway associated metabolite sets (*currently contains 88 entries*);
- Disease associated metabolite sets (reported in blood) (*currently contains 416 entries*);
- Disease associated metabolite sets (reported in urine) (*currently contains 346 entries*)
- Disease associated metabolite sets (reported in CSF) (*currently contains 124 entries*)
- Metabolite sets associated with SNPs (*currently contains 4500 entries*)
- Predicted metabolite sets based on computational enzyme knockout model (*currently contains 912 entries*)
- Metabolite sets based on locations (*currently contains 57 entries*)

In addition, MSEA also allows user-defined metabolite sets to be uploaded to perform enrichment analysis on arbitrary groups of compounds which researchers want to test. The metabolite set library is simply a two-column comma separated text file with the first column for metabolite set names and the second column for its comound names (**must use HMDB compound name**) separated by "; ". Please note, the built-in libraries are mainly from human studies. The functional grouping of metabolites may not be valid. Therefore, for data from subjects other than human being, users are suggested to upload their self-defined metabolite set libraries for enrichment analysis.

6 Enrichment Analysis

Over Representation Analysis (ORA) is performed when a list of compound names is provided. The list of compound list can be obtained through conventional feature selection methods, or from a clustering algorithm, or from the compounds with abnormal concentrations detected in SSP, to investigate if some biologically meaningful patterns can be identified.

ORA was implemented using the *hypergeometric test* to evaluate whether a particular metabolite set is represented more than expected by chance within the given compound list. One-tailed p values are provided after adjusting for multiple testing. **Figure 2** below summarizes the result.

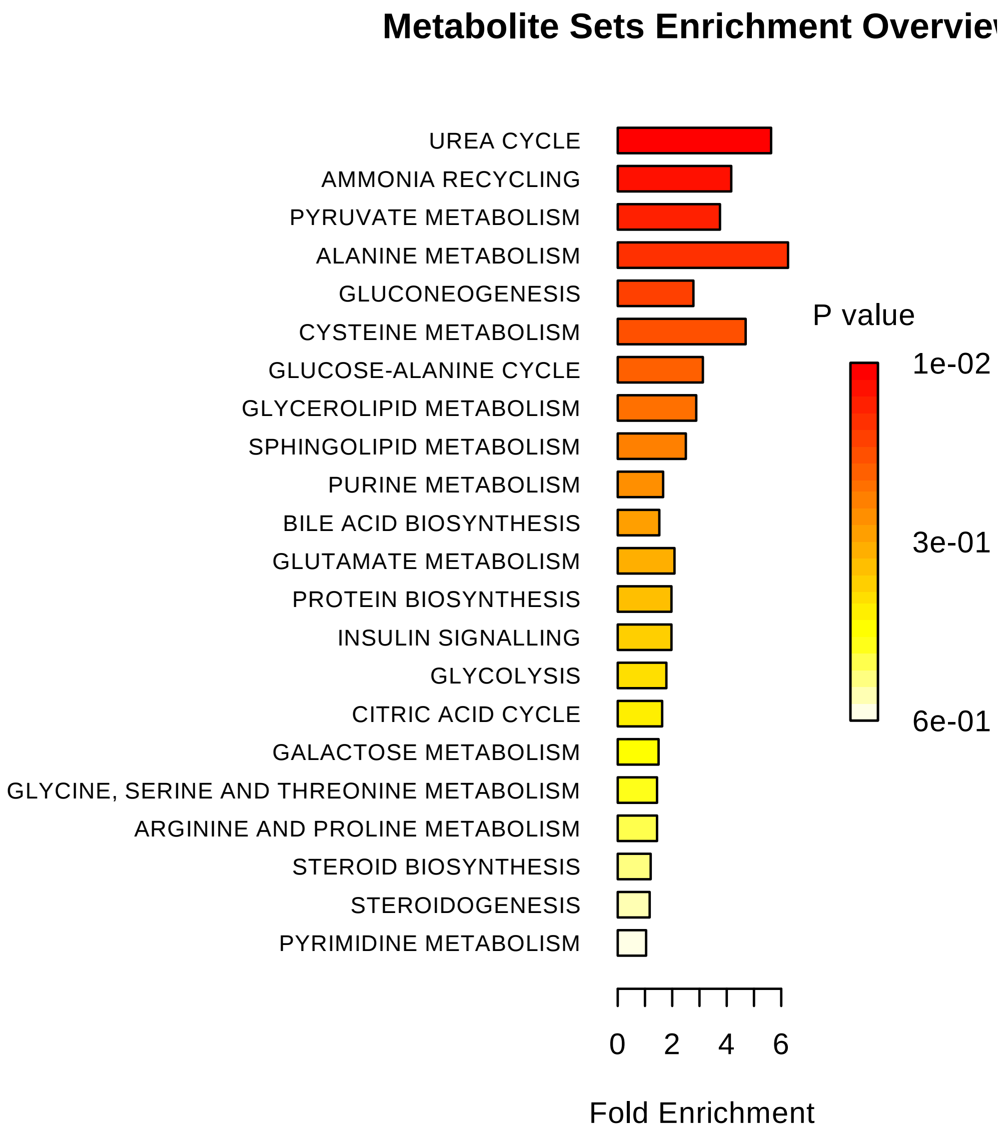

Figure 1: Summary Plot for Over Representation Analysis (ORA)

Table 2: Result from Over Representation Analysis

	total	expected	hits	Raw p	Holm p	FDR
UREA CYCLE	20	0.53	3	1.40E-02	1.00E+00	1.00E+00
AMMONIA RECYCLING	18	0.48	2	8.03E-02	1.00E+00	1.00E+00
PYRUVATE METABOLISM	20	0.53	2	9.65E-02	1.00E+00	1.00E+00
ALANINE METABOLISM	6	0.16	1	1.50E-01	1.00E+00	1.00E+00
GLUCONEOGENESIS	27	0.72	2	1.60E-01	1.00E+00	1.00E+00
CYSTEINE METABOLISM	8	0.21	1	1.95E-01	1.00E+00	1.00E+00
GLUCOSE-ALANINE CYCLE	12	0.32	1	2.79E-01	1.00E+00	1.00E+00
GLYCEROLIPID METABOLISM	13	0.35	1	2.98E-01	1.00E+00	1.00E+00
SPHINGOLIPID METABOLISM	15	0.40	1	3.36E-01	1.00E+00	1.00E+00
PURINE METABOLISM	45	1.20	2	3.40E-01	1.00E+00	1.00E+00
BILE ACID BIOSYNTHESIS	49	1.31	2	3.80E-01	1.00E+00	1.00E+00
GLUTAMATE METABOLISM	18	0.48	1	3.88E-01	1.00E+00	1.00E+00
PROTEIN BIOSYNTHESIS	19	0.51	1	4.05E-01	1.00E+00	1.00E+00
INSULIN SIGNALLING	19	0.51	1	4.05E-01	1.00E+00	1.00E+00
GLYCOLYSIS	21	0.56	1	4.37E-01	1.00E+00	1.00E+00
CITRIC ACID CYCLE	23	0.61	1	4.68E-01	1.00E+00	1.00E+00
GALACTOSE METABOLISM	25	0.67	1	4.96E-01	1.00E+00	1.00E+00
GLYCINE, SERINE AND THREONINE METABOLISM	26	0.69	1	5.10E-01	1.00E+00	1.00E+00
ARGININE AND PROLINE METABOLISM	26	0.69	1	5.10E-01	1.00E+00	1.00E+00
STEROID BIOSYNTHESIS	31	0.83	1	5.74E-01	1.00E+00	1.00E+00
STEROIDOGENESIS	32	0.85	1	5.86E-01	1.00E+00	1.00E+00
PYRIMIDINE METABOLISM	36	0.96	1	6.30E-01	1.00E+00	1.00E+00

The report was generated on Fri Jun 3 11:58:23 2016 with R version 3.2.2 (2015-08-14). Thank you for using MetaboAnalyst! For suggestions and feedback please contact Jeff Xia (*jeff.xia@mcgill.ca*).
