# Supplementary material for: New Biochemical Insights into the Mechanisms of Pulmonary Arterial Hypertension in Humans
Source: PLoS One. 2016 Aug 3;11(8):e0160505. doi: 10.1371/journal.pone.0160505 (PMC4972307; doi:10.1371/journal.pone.0160505)
Supplement: S1 Table — Clinical characteristics of participants. (PDF) [file pone.0160505.s003.pdf]

S1 Table. Study cohort. Clinical characteristics of participants.

STUDY COHORT	PAH	CONTROL
n,%	20 (100)	20 (100)
**Age**, years	51 (15)	50 (15)
**Sex** (women)	15 (75)	15 (75)
**BMI**, Kg/m^2	26 (4)	25 (4)
**PAH aetiology**		-
Idiopathic	8 (40)	-
SSc	6 (30)	-
Other connective tissue disease	2 (10)	-
Congenital	1 (5)	-
HIV	1 (5)	-
Porto-pulmonar	1 (5)	-
Squistosomiasis	1 (5)	-
**FC-WHO** I/II/III/IV (n, %)	6 (30) / 5 (25) / 8 (40) / 1 (5)	-
**Smoking habit** (n,%)		
Never smoker	11 (55)	15 (75)
Former smoker	8 (40)	5 (25)
Current smoker	1 (5)	0
**Cumulative dose**, pack/year	113 (23)	2 (8)
**Comorbidities**		
Ischemic cardiopathy	2 (10)	0
Right heart failure	0	0
Long-term oxygen	6 (30)	0
Systemic hypertension	7 (35)	3 (15)
Renal failure	2 (10)	0
Diabetes	1 (5)	1 (5)
Dyslipidaemia	7 (35)	1 (5)
Hepatitis	1 (5)	0
HIV	1 (5)	0
**Lung Function Test**		
FEV1, L	2.4 (0.76)	3.1 (0.65)
FEV1, %	82 (21)	103 (13)
FVC, L	3.29 (0.95)	3.85 (0.76)
FVC, %	89 (19)	100 (12)
Ratio FEV1/FVC	72(10)	81 (7)
TLC, %	90 (18)	104 (11)
RV, %	99 (26)	110 (20)
DLCO, %	53 (23)	86 (13)
KCO, %	59 (22)	84 (14)
**Gas exchange (FiO2 0.21)**		
PaO2, mmHg	74 (16)	-
PaCO2, mmHg	33 (4)	-
pH	7.44 (0.02)	-
**6MWT,** m	425 (136)	-
**Echocardiography**		
Tricuspid Insufficiency		
No	1 (5)	13 (65)
Mild	8 (40)	7 (35)
Moderate	4 (20)	0
Severe	4 (20)	0
VRT, m/s	3.56 (1.35)	-
Pericardial effusion (n,%)	2 (10)	0
sPAP, mmHg	(n=14) 74 (21)	(n=7) 25 (3)
LVEF, %	62 (6)	63 (3)
Diastolic dysfunction		
No	9 (45)	18 (90)

Mild	6 (30)	2 (10)
Mitral insufficiency		
No	9 (45)	17 (85)
Mild	6 (30)	3 (15)
Moderate	0	0
**Right heart catheterization**		
sPAP, mmHg	71 (21)	-
dPAP, mmHg	32 (11)	-
mPAP, mmHg	45 (14)	-
Cardiac output, L/min	3.94 (1.08)	-
Cardiac index, L/min/m^2	2.28 (0.60)	-
RAP, mmHg	8 (5)	-
PWAP, mmHg	8 (4)	-
PVR, dyn/s/cm^5	838 (401)	-
SvO2, %	64 (10)	-
TRVP positive (n,%)	7 (35)	-
**BNP (n=11)**	242 (349)	20 (13)
**Blood Tests**		
PCR, mg/dL	0.56 (0.66)	0.24 (0.32)
Glucose, mg/dL	95 (29)	96 (25)
Urea, mg/dL	21.53 (10.78)	15.55 (3.04)
Creatinine, mg/dL	0.97 (0.31)	0.75 (0.14)
Uric acid, mg/dL	6.48 (2.09)	4.46 (1.65)
Cholesterol, mg/dL	180 (47)	190 (30)
HDL cholesterol, mg/dL	82 (16)	63 (19)
LDL cholesterol, mg/dL	102 (36)	103 (30)
Triglycerides, mg/dL	123 (73)	95 (56)
Aspartate aminotransferase (AST), UI/L	31 (1)	20 (3)
Alanine aminotransferase (ALT), UI/L	23 (11)	20 (5)
Gamma glutamyl transpeptidase (GGT), UI/L	71 (15)	16 (11)
Alkaline phosphatase (ALP), UI/L	208 (142)	138 (33)
Creatine Kinase (CK), UI/L	88 (63)	(n=6) 76 (60)
Bilirubin, mg/dL	0.85 (0.57)	0.73 (0.24)
Albumin, g/L	43 (5)	46 (7)
Total proteins, g/L	74 (7)	76 (17)
Sodium, mEq/L	141 (2)	144 (10)
Iron, μg/dL	58 (18)	(n=6) 70 (55)
Leucocytes, 10^9/L	7.40 (3.69)	6.72 (1.39)
Haemoglobin, g/L	133 (18)	135 (15)
Haematocrit, L/L	42 (5)	41 (4)
Platelets, 10^9/L	237 (93)	262 (65)
Fibrinogen, g/L	3.61 (1.42)	3.34 (0.52)

Legend: Data are presented as mean (±SD) for quantitative variables, and count (percentage) for discrete variables.
