# Supplementary material for: New Biochemical Insights into the Mechanisms of Pulmonary Arterial Hypertension in Humans
Source: PLoS One. 2016 Aug 3;11(8):e0160505. doi: 10.1371/journal.pone.0160505 (PMC4972307; doi:10.1371/journal.pone.0160505)
Supplement: S2 Table — Clinical characteristics of participants. (PDF) [file pone.0160505.s004.pdf]

S2 Table. Validation cohort. Clinical characteristics of participants.

VALIDATION COHORT	PAH	CONTROL
n,%	20 (100)	12 (100)
**Age**, years	51 (16)	50 (16)
**Sex** (women)	16 (80)	9 (75)
**BMI**, Kg/m^2	26 (4)	25 (4)
**PAH aetiology**		
Idiopathic	7 (35)	-
SSc	7 (35)	-
Other connective tissue disease	2 (10)	-
Congenital	1 (5)	-
HIV	1 (5)	-
Porto-pulmonar	1 (5)	-
Squistosomiasis	1 (5)	-
**FC-WHO** I/II/III/IV (n, %)	2 (10) / 11 (55) / 6 (30) / 1 (5)	-
**Smoking habit** (n,%)		
Never smoker	13 (65)	9 (75)
Former smoker	6 (30)	3 (25)
Current smoker	1 (5)	0
**Cumulative dose**, pack/year	12 (23)	2 (6)
**Comorbidities**		
Ischemic cardiopathy	1 (5)	0
Right heart failure	3 (15)	0
Long-term oxygen	6 (30)	0
Systemic hypertension	4 (20)	11 (92)
Renal failure	0	0
Diabetes	2 (10)	0
Dyslipidaemia	4 (20)	2 (17)
Hepatitis	0	0
HIV	1 (5)	0
**Lung Function Test**		
FEV1, L	2.21 (0.64)	3.12 (0.53)
FEV1, %	79 (14)	112 (10)
FVC, L	2.93 (0.85)	3.88 (0.59)
FVC, %	82 (15)	110 (13)
Ratio FEV1/FVC	76 (9)	80 (6)
TLC, %	88 (15)	108 (10)
RV, %	102 (24)	106 (20)
DLCO, %	47 (20)	101 (15)
KCO, %	59 (23)	95 (21)
**Gas exchange (FiO2 0.21)**		
PaO2, mmHg	73 (17)	-
PaCO2, mmHg	31 (4)	-
pH	7.44 (0.04)	-
**6MWT,** m	445 (91)	-
**Echocardiography**		
Tricuspid Insufficiency		
No	1 (5)	8 (67)
Mild	3 (15)	3 (25)
Moderate	5 (25)	0
Severe	3 (15)	0
VRT, m/s	3.61 (0.54)	-
Pericardial effusion (n,%)	4 (20)	0
sPAP, mmHg	(n=13) 70 (16)	(n=3) 27 (5)
LVEF, %	63 (5)	64 (3)
Diastolic dysfunction	(n=13)	
No	11 (55)	9 (75)

Mild	2 (10)	2 (17)
Mitral insufficiency	(n=13)	(n=11)
No	12 (60)	11 (92)
Mild	0	
Moderate	1 (5)	
**Right heart catheterization**		
sPAP, mmHg	71 (18)	-
dPAP, mmHg	28 (7)	-
mPAP, mmHg	44 (10)	-
Cardiac output, L/min	3.83 (1.18)	-
Cardiac index, L/min/m^2	2.34 (0.76)	-
RAP, mmHg	9 (7)	-
PWAP, mmHg	7 (3)	-
PVR, dyn/s/cm^5	825 (350)	-
SvO2, %	66 (9)	-
TRVP positive (n,%)	4 (20)	-
**BNP (n=11)**	255 (475)	18 (16)
**Blood Tests**		
PCR, mg/dL	0.75 (0.81)	0.18 (0.21)
Glucose, mg/dL	94 (18)	88 (9)
Urea, mg/dL	21.65 (13.83)	14.83 (4.71)
Creatinine, mg/dL	0.86 (0.28)	0.78 (0.17)
Uric acid, mg/dL	6.67 (2.73)	4.65 (1.88)
Cholesterol, mg/dL	160 (98)	206 (28)
HDL cholesterol, mg/dL	48 (15)	58 (15)
LDL cholesterol, mg/dL	95 (25)	86 (44)
Triglycerides, mg/dL	101 (67)	86 (44)
Aspartate aminotransferase (AST), UI/L	26 (8)	25 (6)
Alanine aminotransferase (ALT), UI/L	23 (14)	24 (15)
Gamma glutamyl transpeptidase (GGT), UI/L	74 (68)	24 (16)
Alkaline phosphatase (ALP), UI/L	202 (73)	154 (45)
Creatine Kinase (CK), UI/L	92 (92)	81 (28)
Bilirubin, mg/dL	0.87 (0.56)	0.67 (0.23)
Albumin, g/L	43 (4)	44 (2)
Total proteins, g/L	71 (8)	73 (3)
Sodium, mEq/L	140 (4)	141 (1)
Iron, μg/dL	54 (27)	100 (44)
Leucocytes, 10^9/L	6.72 (1.92)	5.59 (1.1)
Haemoglobin, g/L	141 (15)	137 (11)
Haematocrit, L/L	41 (11)	41 (3)
Platelets, 10^9/L	208 (80)	268 (70)
Fibrinogen, g/L	4.14 (0.93)	3.37 (0.54)

Legend: Data are presented as mean (±SD) for quantitative variables, and count (percentage) for discrete variables.
